# RNAi down-regulation of *cinnamate-4-hydroxylase* increases artemisinin biosynthesis in *Artemisia annua*

**DOI:** 10.1038/srep26458

**Published:** 2016-05-25

**Authors:** Ritesh Kumar, Divya Vashisth, Amita Misra, Md Qussen Akhtar, Syed Uzma Jalil, Karuna Shanker, Madan Mohan Gupta, Prashant Kumar Rout, Anil Kumar Gupta, Ajit Kumar Shasany

**Affiliations:** 1 Biotechnology Division, CSIR-Central Institute of Medicinal and Aromatic Plants, P.O. CIMAP, Lucknow-226015, U.P., India; 2 Analytical Chemistry Division, CSIR-Central Institute of Medicinal and Aromatic Plants, P.O. CIMAP, Lucknow-226015, U.P., India; 3 Chemical Sciences Division, CSIR-Central Institute of Medicinal and Aromatic Plants, P.O. CIMAP, Lucknow-226015, U.P., India; 4 Genetics and Plant Breeding Division, CSIR-Central Institute of Medicinal and Aromatic Plants, P.O. CIMAP, Lucknow-226015, U.P., India

## Abstract

*Cinnamate-4-hydroxylase* (C4H) converts *trans-*cinnamic acid (CA) to *p*-coumaric acid (COA) in the phenylpropanoid/lignin biosynthesis pathway. Earlier we reported increased expression of *AaCYP71AV1* (an important gene of artemisinin biosynthesis pathway) caused by CA treatment in *Artemisia annua*. Hence, *AaC4H* gene was identified, cloned, characterized and silenced in *A. annua* with the assumption that the elevated internal CA due to knock down may increase the artemisinin yield. Accumulation of *trans*-cinnamic acid in the plant due to *AaC4H* knockdown was accompanied with the reduction of *p*-coumaric acid, total phenolics, anthocyanin, cinnamate-4-hydroxylase (C4H) and phenylalanine ammonia lyase (PAL) activities but increase in salicylic acid (SA) and artemisinin. Interestingly, feeding *trans-*cinnamic acid to the RNAi line increased the level of artemisinin along with benzoic (BA) and SA with no effect on the downstream metabolites *p*-coumaric acid, coniferylaldehyde and sinapaldehyde, whereas *p*-coumaric acid feeding increased the content of downstream coniferylaldehyde and sinapaldehyde with no effect on BA, SA, *trans-*cinnamic acid or artemisinin. SA is reported earlier to be inducing the artemisinin yield. This report demonstrates the link between the phenylpropanoid/lignin pathway with artemisinin pathway through SA, triggered by accumulation of *trans-*cinnamic acid because of the blockage at C4H.

The medicinal plant *Artemisia annua* L. is well known for its antimalarial principle artemisinin. Because of its importance, this plant is being investigated for its adaptation to abiotic stress with the ultimate desire to increase artemisinin content^1^,^2^. Strong and stout stem bearing more foliage with higher glandular trichome (GT) density, tolerant to wear and tear are the prerequisites for higher artemisinin harvest. The best molecule for strength to the plant is indubitably the lignin. This molecule is programmed not only developmentally to be deposited in specific tissues but also regulated by different biotic and abiotic stresses^3^. The abiotic stress like water deficit leads to increased lignin biosynthesis in the root^4^ and alters the expression levels of lignin biosynthesis genes^5^. It is logical to expect increased lignin and/or phenolics biosynthesis in view of reported increased expression/activity of phenylpropanoid genes/enzymes like phenylalanine ammonia lyase (PAL), cinnamate-4-hydroxylase (C4H), C3H, 4CL, COMT, CCoAOMT, CAD^4^,^6^,^7^,^8^. Lignins are needed for structural support and water transport, and few plant cell types like xylem elements and sclerenchyma accumulate substantial amounts of lignin contributing strength to the cell^9^. Though, sufficient study has been carried out on cell wall composition and architecture, information on GT cell wall is scarce. According to Peterson and Vermeer^10^, all or part of the cell wall may be impregnated with lignin, cutin, suberin *etc* and the data by Marks *et al.*^11^ in *Arabidopsis* supports the existence of lignin in GT cell walls, which is consistent with their high stiffness and strength as part of their defensive roles. The histochemical tests by Hassan and El-Awadi^12^ showed positive reactions to lignin, phenolic, lipid and suberin materials in the outer layer of GTs, while the phenolic substances were detected in the neck and gland cells. Though a comparison cannot be made between *Arabidopsis* and higher plants glandular trichomes, the role of lignin cannot be ruled out in the architecture of the glandular peltate trichomes. This specialized tissue follows a specific pattern of arrangement of cells providing unique architecture to prevent the premature loss of metabolites. Hence, cell walls of this tissue have to maintain a balance between stiffness/rigidity and flexibility to protect as well as to provide enough space for the storage of metabolites. Further, lignin also controls morphology and anatomy of the plant. Most importantly, earlier during the characterization of Cytochrome P450 monooxygenases (CYPs), we observed that the *AaCYP71AV1* transcript of artemisinin biosynthesis pathway overexpressed significantly with *trans-*cinnamic acid treatment^13^. Considering all these aspects, the functional significance of the C4H was investigated in detail and compared in normal as well as RNAi plants.

C4H converts *trans*-cinnamic acid to *p*-coumaric acid and is the first hydroxylation step of lignin, flavonoids and hydroxycinnamic acid ester biosynthetic pathway correlating with lignifications^14^,^15^,^16^,^17^. This is described to be one of the major flux controlling enzymes for lignification in plants^18^. Hence, the effect of *C4H* gene silencing in *A. annua* was functionally investigated for morphology, anatomy, physiology and biochemistry of the plant. The expression patterns, enzyme analysis through heterologous expression in yeast, sequence analysis, subcellular localization of the C4H protein are discussed in relation to lignification and artemisinin biosynthesis. This report also demonstrates experimentally, the link between lignin/phenylpropanoid and artemisinin biosynthesis pathway at the level of *trans*-cinnamic acid for the first time, indicating the diversion of carbon flux of one pathway for the biosynthesis of signal molecule inducing the biosynthesis of commercially and medicinally important molecule of a different pathway.

## Results

### Expression profile of *AaC4H* in response to abiotic stresses

**Figure 1:**
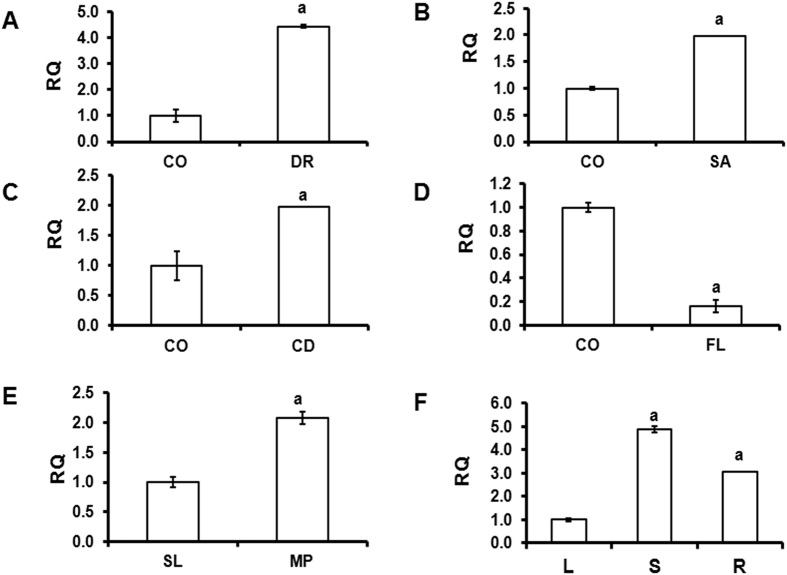
Expression pattern of *AaC4H.* (**A**) Quantitative expression of *AaC4H* in the leaf during drought stress (DR); (**B**) salt stress (SA); (**C**) cold stress (CD) and (**D**) flooding stress (FL) compared to control (CO). (**E**) Quantitative expression at seedling and mature leaf stage (S: 20 days old seedling and L: mature leaf from 150 days old plant). (**F**) Quantitative expression of *AaC4H* in different tissues (L: Leaf, S: Stem, R: Root); Y-axis represents relative quantity (RQ) value. Data are mean ± standard deviation (SD) of 3–5 biological replicates.

Earlier we have isolated a full length C4H gene (GU318226) from Artemisia annua trichome rich tissue^13^. The expression of *AaC4H* significantly increased during drought (Fig. 1A), salt (Fig. 1B) and cold (Fig. 1C) stress but decreased significantly during flooding (Fig. 1D). Quantitative real-time PCR indicated more than 2 fold increase in transcript level in the mature leaf (150 days after sowing) compared to seedlings (20 days after sowing) (Fig. 1E). In the mature plant the expression of *AaC4H* was higher in the stem and root compared to the leaf (Fig. 1F).

### Lignin content of GT and leaf in relation to *AaC4H* expression and artemisinin content

**Figure 2:**
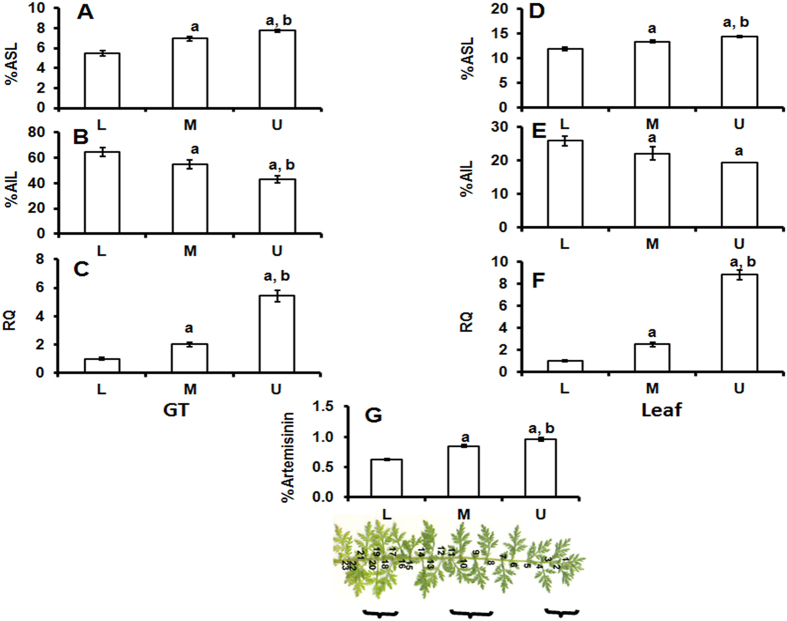
*AaC4H* transcript abundance and lignin content in the GT enriched tissue (**A–C**) isolated from leaf (**D–F**) at lower (L), middle (M) and upper (U) region of the branch as depicted in H. Comparison of acid soluble lignin (ASL) content (**A,D**) and acid insoluble lignin (**B,E**) content, quantitative expression levels of *AaC4H* (**C,F**) and artemisinin content of leaf (**G**). Y-axis represents relative quantity equilibrating the expression at lower region as 1 RQ. Data represent mean ± Standard deviation of 3–5 biological replicates.

The acid soluble lignin (ASL) fraction of the GTs present at upper level of the leaves was higher compared to the middle and lower level leaves (Fig. 2A) whereas the insoluble or Klason lignin content in the GTs of upper level leaves is less and increases as the leaves turn older (Fig. 2B). Similar trend was obtained for the acid soluble and insoluble lignin contents of leaves at different levels (Fig. 2D,E). In the GT enriched fraction as well as in the leaves, higher expression of this gene was detected in the GTs and leaves of upper level followed by middle and lower level (Fig. 2C,F). The artemisinin content of leaves at different level correlated well with the ASL content and expression pattern of *AaC4H* (Fig. 2G).

### Heterologous expression of CPR and *AaC4H* cDNA in yeast and enzyme assay

About 3–4 fold higher activity was observed in the microsomes expressing AaCPR compared to the microsomes of yeast transformed with empty vector only (Figure S1). The AaCPR (80 kDa) was also identified in the gel by immunodetection through western blotting using anti-c-Myc antibody (Figure S1). The isolated microsomal fraction (containing AaC4H and AaCPR) was incubated with *trans*-cinnamic acid for enzyme assay. HPLC analysis of the reaction showed a new peak at retention time of ~5 min along with the substrate peak at retention time of ~12 min. The peak observed at retention time of ~5 min was confirmed as *p*-coumaric acid by UV absorbance and authentic reference standard (Figure S2). The K_m_ value for *trans*-cinnamic acid was determined to be 6.4 μM and V_max_ was calculated to be 0.029 mM min^−1^ mg^−1^.

### Subcellular localization of AaC4H

**Figure 3:**
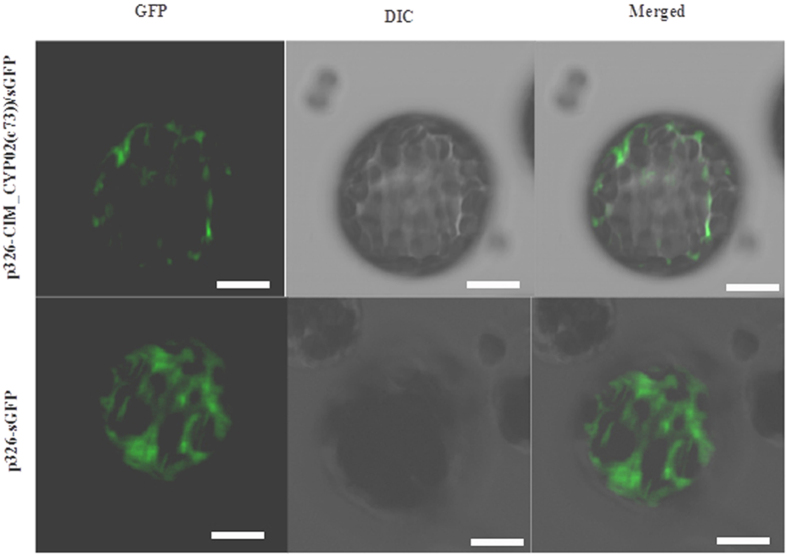
Subcellular localization of AaC4H protein. The full-length coding region of AaC4H was fused to the GFP reporter gene in the vector p326-sGFP to produce the construct p326-AaC4H/sGFP. Expression of p326-AaC4H/sGFP and GFP alone was driven by the 35S promoter in *Arabidopsis* protoplasts, examined by confocal laser scanning microscopy. GFP image (Left), Differential interference contrast (DIC) images (Center), merged image of GFP and DIC (Right). (Scale bars = 50 μm).

GFP fluorescence for AaC4H full-length coding region GFP fusion was observed as a diffused signal predictably in the endoplasmic reticulum (ER) (Fig. 3). Expression of control GFP construct was localized in the cytoplasm. The inference on expression pattern in ER was drawn by comparing earlier published reports^17^.

### Transgenic RNAi plant for *AaC4H*

**Figure 4:**
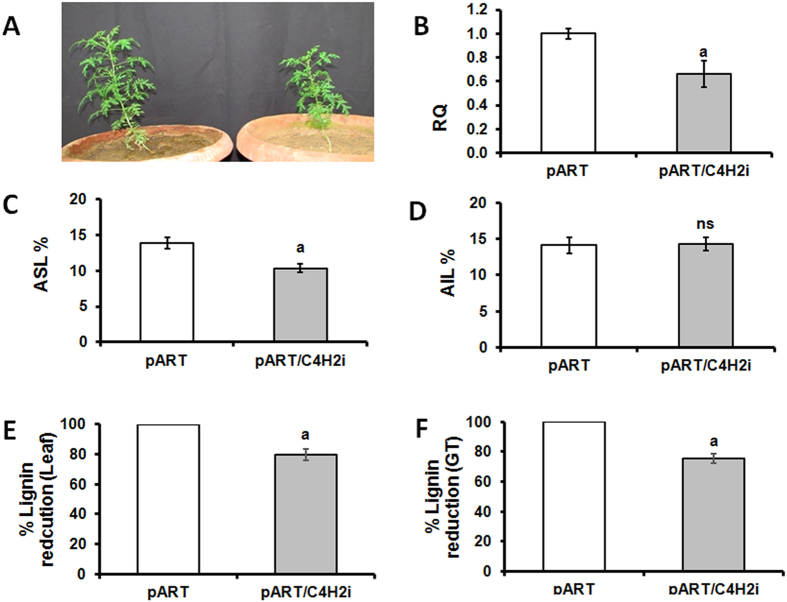
Comparison between vector transformed (pART) and RNAi plants (pART/C4H2i) for *AaC4H* gene. Reduced growth of RNAi plants (after 6 weeks) (**A**); quantitative expression (**B**); percent (dry weight) acid insoluble lignin (AIL) (**C**); acid insoluble lignin (AIL) (**D**); percent reduction in total lignin of leaf (**E**) and GTs (**F**). Data represent mean ± Standard deviation of 3–5 biological replicates.

**Figure 5:**
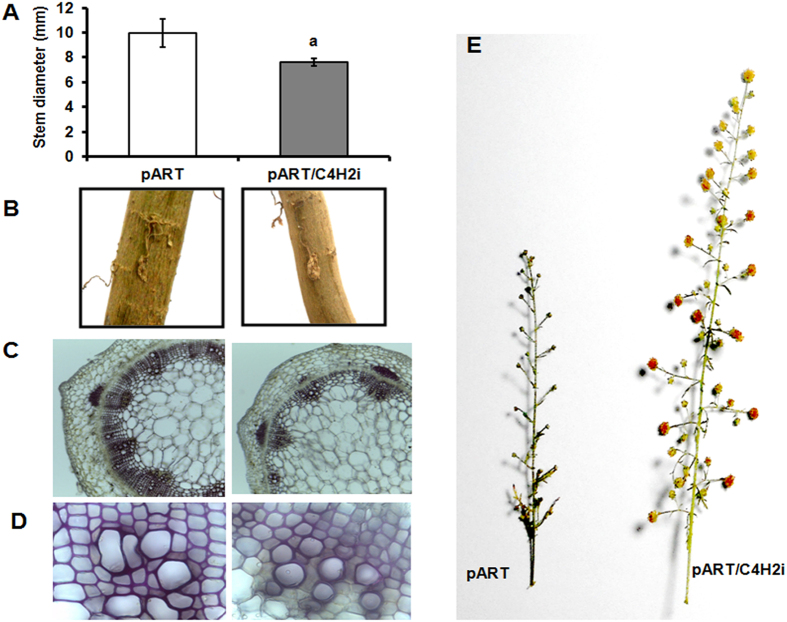
Morphological and anatomical differences. Decrease in stem diameter at the lowest internode (**A**); thin stem of RNAi plant (**B**); depressed xylem development (**C,D**); bigger inflorescence (**E**) of the *AaC4H* knock down plant. Data represent mean ± Standard deviation of 3–5 biological replicates.

**Figure 6:**
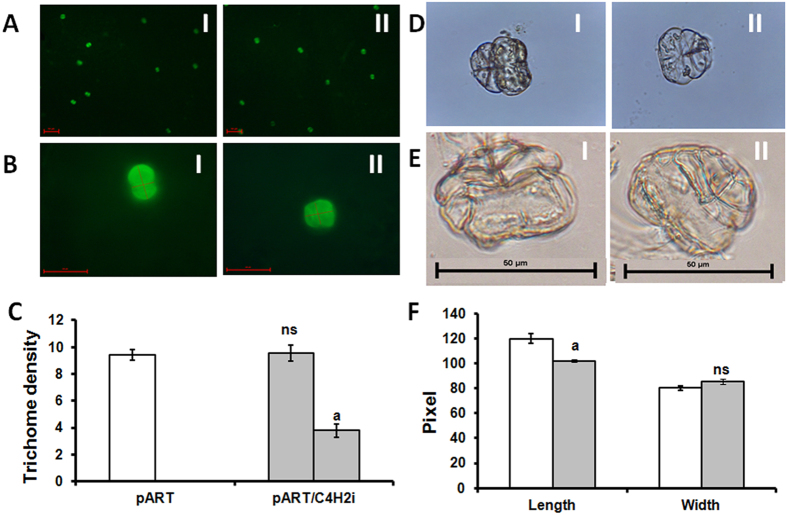
GT density and morphology. Photograph showing density of GTs in the microscopic field (**A**); morphological change in GT (**B**); average GT density and number with changed morphology (**C–E**); comparison of length and width of trichomes (**F**). Left gray column in C indicates no significant change in the GT density where as the right gray column shows 40% of GTs have altered morphology with reduced length. Data represent mean ± Standard deviation of 3–5 biological replicates. Blank column: pART and gray column: pART/C4H2i.

**Figure 7:**
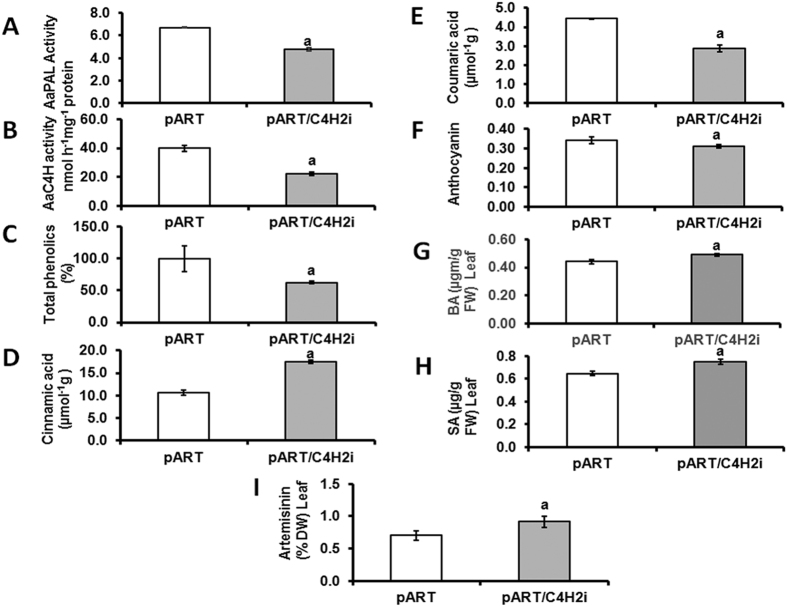
Analysis of enzyme activity and metabolites. AaPAL activity (**A**); AaC4H activity (**B**); total phenolics (**C**); levels of *trans*-cinnamic acid (**D**); *p*-coumaric acid (**E**); anthocyanin (**F**); benzoic acid (**G**); salicylic acid (**H**) and artemisinin (**I**). Data represent mean ± Standard deviation of 3–5 biological replicates.

From the amplification of NPTII gene and the integrated intron (pHANNIBAL vector), two independent RNAi lines could be screened (pART/C4H1i and pART/C4H2i). Both the lines showed similar morphology and expression patterns and hence one line (pART/C4H2i) was carried forward for detailed analysis. This line was investigated for the boarder sequences at the position of integration of the RNAi cassette into the plant genome and the sequences did not show similarities with any regulatory or structural gene(s) of *A. annua* (Table S2). The RNAi line was observed to be slower in growth compared to the vector transformed plants (Fig. 4A) accompanied with reduced expression level for the *AaC4H* gene (Fig. 4B). The acid soluble lignin fraction in the leaves of RNAi plant was significantly lower compared to the vector transformed plant (Fig. 4C) though the difference between acid insoluble fractions was not significant (Fig. 4D). A reduction of total lignin of leaf (Fig. 4E) and GT (Fig. 4F) was observed. A visible reduction in the thickness of stem (Fig. 5A; Fig. 5B) due to impact on xylem organisation and development (Fig. 5C,D) was detected in the stem section of RNAi line compared to the vector transformed plants. The phloroglucinol staining was less intense in the xylem of the RNAi line (Fig. 5D). Interestingly, the RNAi transgenic line for *AaC4H* flowered one month earlier with bigger inflorescences (Fig. 5E). Although no significant difference was observed for the GT density (Fig. 6A,C) in the leaf of the RNAi line, about 40% of the GTs were significantly smaller in length (Fig. 6B,C,F) with less intense lignin specific stain in the cell walls of the trichome tissue (Fig. 6D,E). The difference was more prominent in the stalk as well as the basal cells. In the *AaC4H* silenced RNAi line significant decrease in phenylalanine ammonia lyase (PAL) activity (Fig. 7A) was observed with the accumulation of *trans-*cinnamic acid (Fig. 7B). But, a similar decrease in AaC4H activity (Fig. 7C) was accompanied with a decrease in *p*-coumaric acid (Fig. 7D). In addition, there was a decrease in total phenolics (Fig. 7E) and anthocyanin content in the RNAi line for *AaC4H* (Fig. 7F). Interestingly, an increase in salicylic acid (SA) (Fig. 7G) and artemisinin content was observed in the RNAi line knocked down for *AaC4H* gene (Fig. 7H).

### Feeding *trans*-cinnamic and *p*-coumaric acid to RNAi line and metabolite compensation

**Figure 8:**
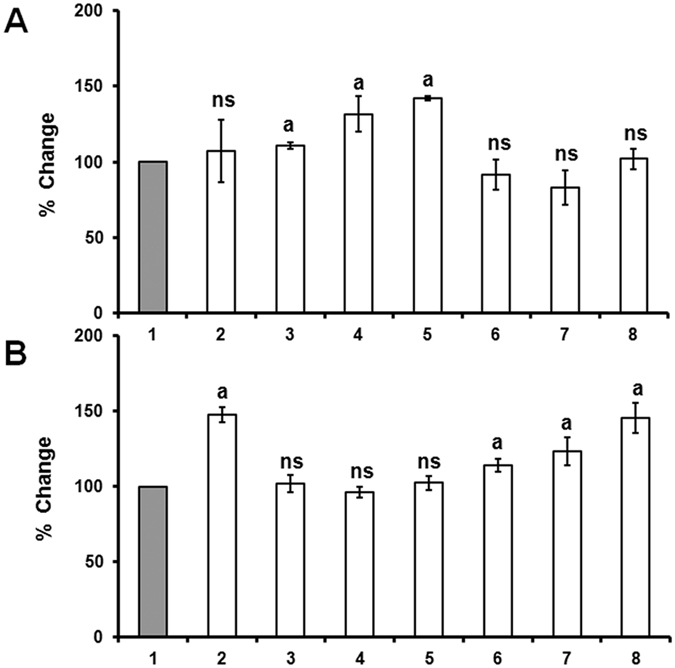
Change in the level of *trans*-cinnamic acid (2); *p*-coumaric acid (3); coniferylaldehyde (4); sinapaldehyde (5); benzoic acid (6); salicylic acid (7) and artemisinin (8) in the leaf of RNAi plant when fed with *p*-coumaric acid (**A**) and *trans*-cinnamic acid (**B**) compared to the twigs without feeding. Data represent mean ± Standard deviation of 3–5 biological replicates.

When the RNAi knockdown line pART/C4H2i was fed with *p*-coumaric acid, a significant increase in *p*-coumaric acid, coniferaldehyde and sinapaldehyde was detected but no significant change in the content of *trans*-cinnamic acid, benzoic acid, salicylic acid and artemisinin was observed (Fig. 8A). Similarly, feeding *trans-*cinnamic acid increased the contents of *trans-*cinnamic acid, benzoic acid, salicylic acid and artemisinin significantly, without any change in *p*-coumaric acid, coniferaldehyde and sinapaldehyde contents (Fig. 8B).

## Discussion

Commercially and therapeutically important artemisinin is biosynthesized and stored in GTs of flowers and on both the surfaces of leaves^19^. Hence the GTs are crucial to the yield of artemisinin. According to Lommen *et al.*^20^ GT density is highest at the maximum size of leaves, after which density decreases rapidly, suggesting the rupture over time in the older leaves. Similar decrease in the GT number in *Mentha arvensis* from upper expanding young, to the lower level leaves proceeding for senescence is reported^21^. Duke and Paul^19^ also indicated the breakage of subcuticular sac in many mature glands releasing secondary metabolites. The strength of GTs and the cuticle depends upon the nature and composition of the protective walls of the tissue. Presence of lignin has been reported in the cell walls of GTs^12^ but never being indicated for their architecture and protection of the stored metabolites. In addition, lignin biosynthesis has never been correlated to the artemisinin biosynthesis pathway. Hence, the second gene in the lignin biosynthesis pathway (*C4H*) was isolated and functionally characterized in detail in *Artemisia annua*.

### Stress response and lignin biosynthesis

The enzyme C4H is described to be highly active during drought stress^8^. Salinity and cold stress^22^ also induce the *C4H* expression in addition to some other phenylpropanoid pathway genes. Flooding stress represses *C4H* along with other phenylpropanoid genes in poplar altering the lignification^23^. The association of higher lignin and developed xylem with increased *C4H* expression is logical and down-regulation of this enzyme in alfalfa reportedly decrease the total lignin contents^24^. Similar trend of expression patterns for *AaC4H* was observed in relation to different stresses in this investigation. Though the lignin pathway is modulated positively during stress; artemisinin content was reported to be negatively modulated by prolonged water deficit but positively controlled by the growth and development^2^ indicating probable diversion of carbon flux towards lignin and other phenyl propanoid in *A. annua*.

### Involvement of *AaC4H* in lignin biosynthesis of GTs

Amount of lignin varies in different parts and with the age of plant. *AaC4H* also showed difference in expression at different developmental stages. So, it may be reasonable to predict that the increased level of ASL in the upper level GTs (with high *AaC4H* expression) may help in providing better protection for the storage of artemisinin. Whereas, in older leaves the matured GTs with less ASL and high acid insoluble lignin (AIL) (brittle) may break to release the stored artemisinin with little disturbance, decreasing the yield. Yasuda *et al.*^25^ reported higher ASL content of syringyl lignin-rich wood and the higher reactivity of the syringyl nucleus to sulphuric acid than the guaiacyl nucleus. This also suggests an important relation between ASL and syringyl lignin. Higher *AaC4H* gene expression, ASL content (hence, S lignin content) observed in the growing GTs of upper level leaves which decreased towards the lower level matured leaf GTs, suggest the involvement of *AaC4H* in lignin biosynthesis of developing GTs and leaves. Along with this, the leaves of upper level showed higher artemisinin content compared to the leaves at lower level. In the plant *A. annua*, artemisinin concentration is reported to be higher in upper leaves compared to lower in a branch^26^,^27^.

### AaC4H function and localization

The apparent K_m_ of AaC4H for *trans*-cinnamic acid was determined to be 6.41 μM. Similar K_m_ values for *trans*-cinnamic acid varying from 0.7 μM to 8.9 μM is reported earlier^14^,^28^,^29^,^30^,^31^. Subcellular localization of pea seedlings C4H was suggested to be in the endoplasmic reticulum (ER)^32^. GFP fluorescence for p326-AaC4H-sGFP fusions was observed as a diffused signal exclusively in the ER and confirmed by comparing the fluorescence pattern of ER targeted proteins already reported in *Populus* and other plants^17^. Secretory cells of GTs contain extensive endoplasmic reticulum^19^ which is indicated for cell to cell movement of small molecules^33^. This helps the smooth movement of small molecules for conversion to monolignans to be deposited in the cell wall to provide shape and strength essential for architecture and protection of GTs.

### *AaC4H* knock down leads to defects in morphology, anatomy and reduction in lignin content in *A. annua*

Several researchers targeted *C4H* gene for downregulation in tobacco^15^,^16^, alfalfa^34^, *Populus*^35^ and rice^36^. The decreased C4H activity in a protein folding defective *C4H* mutant causes pleiotropic phenotypes, including dwarfism, male sterility and the development of swellings at branch junctions in addition to decreased levels of several different classes of phenylpropanoid end-products, and exhibit reduced lignin deposition and altered lignin monomer content^37^. Interestingly, RNAi transgenic rice plant for C4H was observed to be having lower lignin content without affecting normal field agronomic traits^36^. In case of *A. annua* also, the growth of the *AaC4H* knock down plants were stunted with reduction in lignin content in both leaf and GTs. A significant reduction in ASL was detected in the leaves of *AaC4H* knockdown plants though the AIL content was similar to control. The plants were also having thinner stems bearing bigger inflorescences, flowering one month earlier. In addition, collapsed xylem in the stem was like the earlier observation in *Arabodopsis* C4H mutant *Ref3*^3^,^37^.

### Effect of *AaC4H* knock down on GT and metabolites

In *AaC4H* knockdown plant, though the lignin content was significantly lower compared to the control, the GT density remained same. Water deficit stress positively modulates the *C4H* gene (Fig. 1) but induces a decrease in GT density and size as well^2^. In the present investigation downregulation of *C4H* does not have any effect on GT number. But, about 40% of GTs in the knockdown plant were lesser in length with similar width. Hence, it may be argued that the water stress affects the biogenesis and differentiation of GT which is independent from lignin deposition for strength and architecture through the expression of C4H. The GTs also took less lignin specific stain indicating lower lignin deposition in the RNAi plant. This may also be the cause for decreased strength and rupture during isolation.

**Figure 9:**
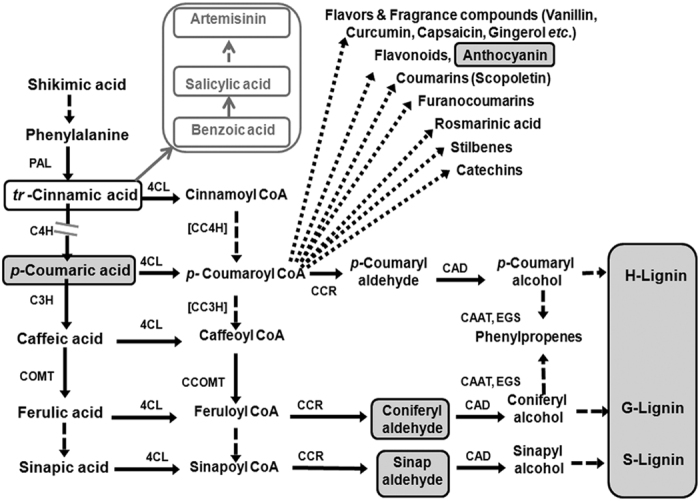
Pathway indicates the modulation of metabolites in the RNAi plant. Accumulation and overproduction of *trans*-cinnamic acid due to *AaC4H* knockdown leads to significant increase in benzoic acid, salicylic acid and artemisinin (represented in empty boxes) and decrease in the content of *p*-coumaric acid, lignin, anthocyanin, coniferylaldehyde and sinapaldehyde (represented in gray boxes). PAL, phenylalanine ammonia lyase; C4H, cinnamate 4-hydroxylase; 4CL, 4-coumarate:CoA ligase; C3H, p-coumarate 3-hydroxylase; COMT, caffeoyl O-methyl transferase; CC4H, cinnamoyl-CoA 4-hydroxylase; CC3H, p-coumaroyl-CoA 3-hydroxylase; CCOMT, caffeoyl-CoA O-methyl transferase; CCR, cinnamoyl-CoA reductase; CAD, cinnamyl alcohol dehydrogenase; CAAT, coniferyl alcohol acetyl transferase; EGS, eugenol (and chavicol) synthase.

Interestingly, increased ASL was related to higher expression of *AaC4H* as well as higher artemisinin in the control plant (without any stress) as observed in the leaf at upper level, where as in the knockdown plant for *AaC4H*, reduced expression was related to decreased ASL and increase in artemisinin. But, during drought stress higher expression of *AaC4H* is observed though artemisinin content is reported to be negatively modulated by the same condition^2^. These results generated the curiosity on the role of *C4H* in modulating artemisinin biosynthesis through a separate mechanism delinking the stresses where reduced GT number will definitely decrease the artemisinin yield. We have earlier reported the overexpression of *AaCYP71AV1* transcript of artemisinin biosynthesis pathway with *trans-*cinnamic acid treatment^13^. Higher expression of *AaC4H* converts the substrate *trans-*cinnamic acid to *p-*coumaric acid in the upper level of leaves of normal plant to be utilized by downstream pathways. This also ensures availability of *trans-*cinnamic acid pool for utilization by other branched pathway. Hence, the balance between availability of *trans-*cinnamic acid inducing artemisinin biosynthetic pathway to produce more artemisinin and the breakage of GTs with reduced strength due to impaired lignin biosynthesis (loss of artemisinin) determines the net artemisinin content in the *AaC4H* knocked down plant. Lignin modified plants with decreased lignin biosynthesis are shown to be altering carbon flow within the phenylpropanoid pathway and indirectly affect the synthesis of other secondary metabolites^38^,^39^. Chemical inactivation of *C4H* also leads to the accumulation of salicylic acid (SA) in elicited cells of tobacco^40^. Plants with down regulated lignin biosynthesis are reported to be having higher levels of SA relative to controls^39^. Reduced flux delivery into phenylpropanoid pathway due to reduced activity of C4H and accumulation of *trans-*cinnamic acid, a feedback modulator of PAL has also been described earlier^15^,^41^. Hence, the leaf of RNAi line accumulated significant amount of *trans-*cinnamic acid with reduced *p-*coumaric acid resulting in decreased activity of PAL and C4H and the accumulated *trans-*cinnamic acid is diverted for the biosynthesis of SA through benzoic acid (BA). Schoch *et al.*^40^ strongly suggested the branching of SA from *trans-*cinnamic acid and not from chorismate (Fig. 9). Pu *et al.*^42^ and Aftab *et al.*^43^ have reported the role of SA activating the artemisinin biosynthesis in *A. annua* by inducing the expression of 3-hydroxy-3-methylglutaryl coenzyme A reductase (HMGR) and amorpha-4,11-diene synthase (ADS) followed by a burst of reactive oxygen species (ROS) and the conversion of dihydroartemisinic acid into artemisinin. Hence, an increase in BA and SA was observed in the *AaC4H* knocked down plant with increased *trans-*cinnamic acid, artemisinin.

### Feeding *trans*-cinnamic acid increases artemisinin content

To prove this further, the twigs of transgenic plants were dipped in *trans-*cinnamic acid overnight and interestingly the level of artemisinin increased further without any effect on the downstream metabolites coniferylaldehyde and sinapaldehyde, which is explainable by the block at *AaC4H* in the RNAi plant. In contrast *p-*coumaric acid treatment increased downstream coniferylaldehyde and sinapaldehyde with no significant difference in the artemisinin content. This confirms the role of *trans-*cinnamic acid in modulating artemisinin biosynthesis. In this investigation an increase in BA and SA was observed when the twigs were treated with *trans-*cinnamic acid compared to the treatment with *p-coumaric* acid. In the normal plant, the density, position and strength of GTs are dominant factor along with normal biosynthesis of *trans-*cinnamic acid, whereas in RNAi plant, density, position and accumulation of more *trans-*cinnamic acid leading to increased SA plays dominant role for increased artemisinin biosynthesis. This confirms the role of *trans-*cinnamic acid in modulating artemisinin biosynthesis through SA pathway and is the first report demonstrating the relationship between the lignin and sesquiterpene biosynthesis experimentally. Ignoring the stunted growth of the RNAi plant with reduced expression of *AaC4H* because of impaired lignin biosynthesis as the construct was under a constitutive promoter (CaMV), the investigation proves the linkage between phenylpropanoid and artemisinin biosynthetic pathways and opens up the possibility to overexpress *trans-*cinnamic acid in the GTs to increase the artemisinin content of the plant. In other words specific overexpression of *trans-*cinnamic acid in the GTs may increase the artemisinin content, avoiding pleiotropic phenotypes due to defective lignin biosynthesis through RNAi.

## Materials and Methods

### Plant material and treatments

*A. annua* var. ‘CIM-Arogya’^44^, from the National Genebank for Medicinal and Aromatic Plants (NGMAP) at CIMAP, was grown in the field during February and August. Leaf samples were collected from 20 and 150 days after sowing for RNA isolation. GT and leaf materials were isolated for lignin and expression analysis from 3 levels of leaves at the tip of the branch till 3^rd^ node, upper level (U); from 8, 9, 10 and 11^th^ nodes, middle level (M); and from 17, 18, 19 and 20^th^ nodes, lower level (L). Mature plants were subjected to different stress treatments. For drought, plants were irrigated well on the first day followed by without irrigation for 10 days in the glass house. Plants were irrigated everyday to maintain the moisture level above the water holding capacity of the soil for water logging (flooding) stress. For salinity, plants were irrigated with 100mM NaCl solution three times during the period of 10 days. Plants were maintained at 4 °C for 10 days in the cold room for cold stress. Samples from all the treatments were collected on 11^th^ day for expression analysis.

### Isolation and cloning of AaC4H and AaCPR in pESC-URA

GTs were isolated from young leaves of *A. annua* by following protocol based on the glass-bead abrasion technique^45^. About 100 mg GT enriched tissue was used for total RNA isolation^46^ and 5 μg RNA was taken to make cDNA using Thermoscript RT PCR System (Invitrogen, USA). Full length *AaC4H* and *AaCPR* genes were isolated and cloned at the MCS1 and MCS2 site of pESC-URA as described by Misra *et al.*^13^.

### Quantitative RT-PCR Analysis of *AaC4H*s

The expression levels of *AaC4H* at various conditions were measured by real-time PCR with SYBR green I chemistry (Applied Biosystems, USA) with specific primers generating single discrete fragment of size about ~150 bp with no primer–dimers following the protocol described by Rastogi *et al.*^47^. All the primer sequences are provided in Table S1.

### Expression of *AaCPR* and *AaC4H*

*Saccharomyces cerevisiae*, YPH501 competent cells were transformed with 1–3 μg of the pESC::CPR and pESC::CPR-C4H plasmids. Expression of AaCPR and AaC4H, and microsome isolation was carried out following the protocol described earlier^13^.

### Western blotting and hybridisation

Western blotting was carried for detection of c-Myc epitope tagged AaCPR protein cloned in pESC-URA vector. Microsomal protein separated by one-dimensional SDS-PAGE was transferred to a 0.45 μm, 7.9 × 10.5 cm nitrocellulose paper, using a blotting unit (Biorad) for a period of 4–5 h at 90 mA, 30V and the protein was detected using anti-cMyc and HRP-linked anti-mouse antibody combination.

### Cytochrome P450 reductase assay

Cytochrome C (bovine heart, Sigma, USA) solution (100 μl, 6.5 mg/ml) in potassium phosphate buffer (50 mM, pH-7.5) was mixed with microsomal protein (0.1 mg) and volume was made up to 950 μl with potassium phosphate buffer (50 mM, pH-7.5). Reaction was started by adding aqueous NADPH solution (50 μl, 9 mg/ml). For the reference sample, water was added instead of NADPH. Cytochrome C was used as an artificial electron acceptor to measure the reductase activity of CPR and reduced cytochrome C was measured at 550 nm for 10 min. The rate of reduction was calculated by an extinction coefficient of 21 mM^−1^cm^−1 ^48.

### Cinnamate-4-hydroxylase assay and subcellular localisation of AaC4H

Total microsomal protein was isolated and reaction for cinnamate-4-hydroxylase activity was carried out following the protocol described by Ro *et al.*^17^. The reaction products were analyzed using HPLC (Shimadzu LC-10) equipped with spherisorb ODS2 column (4.60 × 250 mm, 10 μm) and a photo diode array detector. The mobile phase consisted of acetonitrile: water (containing 1% tri-fluoro acetic acid) 35: 65 with a flow rate of 1 ml/min. HPLC peak specific to *p*-coumaric acid was identified by migration of standard and diagnostic UV absorption spectra at 310 nm. Peak area was used to quantify the product. *K*_*m*_ and *V*_*max*_ were estimated by Lineweaver–Burk plots^49^ from average of 5 replicates. Cellular localization study was performed following the protocol described by Rastogi *et al.*^47^. The open reading frames of AaC4H was fused upstream of GFP in the cloning sites *Xba*I and *Bam*HI (Table S1) of the p326-sGFP vector containing the CaMV 35S promoter and used in the localization study.

### RNAi gene construct and transgenic *A. annua*

RNAi construct was prepared by amplifying the sense and antisense gene fragments using specific primers (Table S1) corresponding to +1 to +456 region of *AaC4H* from ATG codon. These fragments were cloned sequentially on either side of the intron between *Xho*I/*Eco*RI and *Bam*HI/*Hind*III restriction sites under CaMV 35S promoter of pHANNIBAL vector (CSIRO, Australia), respectively, to get the two arms of the hairpin. The complete hairpin cassette was cloned into pART 27 binary vector^50^ using the *Not*I restriction site (pART/*C4Hi*). The binary vector with and without the hairpin cassette was then transformed into GV3103 strain of *Agrobacterium* separately. The transformation method as described earlier^51^ was used to generate transgenic *A. annua* plant expressing the RNAi construct for *AaC4H*. *Agrobacterium* strain GV 3103 containing pART/*C4Hi* construct (200 μl) was inoculated in 40 ml YEP medium containing 50 μg ml^−1^ rifampicin, 40 μg ml^−1^ gentamicin and 50 μg ml^−1^ kanamycin and incubated for overnight at 28 °C (up to OD_600_ 0.4–0.6) and used for transformation. Fully acclimatised plantlets were grown in the greenhouse. For analysis, samples were collected from 4 month old plants. As two independent transgenic plants with similar morphology and expression patterns were obtained (pART/*C4H1i* and pART/*C4H2i*), only pART/*C4H2i* was taken further for characterization. To ascertain the site of integration flanking regions of left and right border of T-DNA were identified using Genome Walker universal kit (Clontech, US) and analyzed by blasting NCBI as well as TrichOME database.

### Lignin and artemisinin extraction and analysis

Lignin content (ASL, AIL and total) was estimated from GTs and the whole leaf using the protocol described by Mann *et al.*^52^. In addition, lignin content was analyzed following the protocol described by Kline *et al.*^53^. Artemisinin extraction and analysis was carried out following the protocol described by Misra *et al.*^13^.

### Total phenolic and anthocyanin estimation

Total phenolic content was estimated according to the method described by Luqman *et al.*^54^ in terms of gallic acid equivalents. Fully expanded fresh leaves were used for measuring the anthocyanin level^55^.

### Analysis of *trans*-cinnamic, *p*-coumaric acid, coniferalehyde and sinapaldehyde

Metabolites were analyzed as described by Proestos and Komaitis^56^. After extraction, filtering and dilution to 500 μl, 10 μl was injected to HPLC (Shimadzu, LCMS-2010 EV). The instrument was equipped with Waters Symmetry C18 column (5 μ, 4.6 mm × 250 mm) and flow rate was maintained at 1 ml/min. Separation was achieved in gradient mode with mobile phase consisting of water with 2% acetic acid (solvent A) and methanol - acetonitrile (Solvent B, 50:50 v/v). The gradient program was set with slight modifications as: 10% B (0–5 min.), 40% B (5–25 min.), 45% B (25–35 min.), 55% B (35–40 min.) and finally 10% B (40–45 min.). Absorbance was monitored at 280 nm for *trans*-cinnamic acid, 320 nm for *p*-coumaric acid, 340 nm for coniferalehyde and sinapaldehyde, and retention time was matched with authentic standards.

### Salicylic acid and benzoic acid estimation

Salicylic acid and benzoic acid were quantified as described by Deng *et al.*^57^. For quantification of SA and BA 1 μl of derivatized sample was injected in GC-MS (Agilent GC-7890A, MS-5977A) equipped with HP-5MS capillary column. Oven was programmed as initial 100 °C for 2 min with an increase of 15 °C/min up to 300 °C and finally 10 min hold at 300 °C. SA and BA was identified and quantified by corresponding standards and their respective mass spectra matched with NIST- Library.

### Histochemical Staining of lignin

Phloroglucinol-HCl reagent (2 volume of 2% w/v phloroglucinol in 95% ethanol and 1 volume of concentrated HCl) was prepared according to Guo *et al.*^58^ and used for lignin staining of stem sections and trichomes isolated from control and transgenic *A. annua* plants. Image was taken at magnification of 10× for stem and 100× for xylem using Leica DM750 compound microscope. GTs were also visualized at 60× using Nikon Eclipse Ti-S fluorescence microscope.

### Fluorescence microscopy of GTs

Leaves of control and transgenic *A. annua* were analyzed on Nikon Eclipse Ti-S fluorescence microscope with filter settings for FITC (λex 480 nm; λem = 535 nm). Photographs were taken at 2 sec exposure time in binning mode. GT density and morphology were analyzed at 20X and 60X magnifications respectively using NIS elements BR software version 4.0.

### Feeding *trans*-cinnamic and *p*-coumaric acid to RNAi line

Leaves from 5 months old RNAi plant were dipped in 100 mM *p*-coumaric/*trans*-cinnamic acid solutions (in 1% methanol) separately for overnight at 30 °C with shaking. Leaves dipped in 1% methanol served as the control treatment. Leaves were washed properly with distilled water after treatment and dried at 37 °C for 2 days. *p*-Coumaric/*trans*-cinnamic acid, coniferaldehyde, sinapaldehyde, benzoic acid, salicylic acid and artemisinin contents were estimated as described earlier.

## Additional Information

**Accession numbers**: Sequence data from this article can be found in the GenBank/EMBL data libraries under accession numbers GU318226 (AaC4H) and JN594507 (AaCPR).

**How to cite this article**: Kumar, R. *et al.* RNAi down-regulation of *cinnamate-4-hydroxylase* increases artemisinin biosynthesis in *Artemisia annua*. *Sci. Rep.* **6**, 26458; doi: 10.1038/srep26458 (2016).

## Supplementary Material

Supplementary Information
