# Supplementary material for: RNAi down-regulation of cinnamate-4-hydroxylase increases artemisinin biosynthesis in Artemisia annua
Source: Sci Rep. 2016 May 25;6:26458. doi: 10.1038/srep26458 (PMC4879530; doi:10.1038/srep26458)
Supplement: Supplementary Information [file srep26458-s1.pdf]

Supplementary Data

RNAi down-regulation of *cinnamate-4-hydroxylase* increases artemisinin biosynthesis in *Artemisia annua*

All author names and affiliations:

Ritesh Kumar[1a], Divya Vashisth[1a], Amita Misra[1], Md Qussen Akhtar[1], Syed Uzma Jalil[1], Karuna Shanker[2], Madan Mohan Gupta[2], Prashant Kumar Rout[3], Anil Kumar Gupta[4], Ajit Kumar Shasany[1]*

[1]Biotechnology Division, CSIR-Central Institute of Medicinal and Aromatic Plants, P.O. CIMAP, Lucknow-226015, U.P., India

[2]Analytical Chemistry Division, CSIR-Central Institute of Medicinal and Aromatic Plants, P.O. CIMAP, Lucknow-226015, U.P., India

[3]Chemical Sciences Division, CSIR-Central Institute of Medicinal and Aromatic Plants, P.O. CIMAP, Lucknow-226015, U.P., India

[4]Genetics and Plant Breeding Division, CSIR-Central Institute of Medicinal and Aromatic Plants, P.O. CIMAP, Lucknow-226015, U.P., India

a. contributed equally

***Corresponding author(s) details:**

Ajit K. Shasany, Biotechnology Division,
CSIR-Central Institute of Medicinal and Aromatic Plants (CSIR),
P.O. CIMAP, Lucknow-226015, U.P., India
Phone: 91-522-2718548
Fax: 91-522-2342666
Email: ak.shasany@cimap.res.in

Corresponding author email

Email: ak.shasany@cimap.res.in

Table S1: Primers used in this investigation

S No	Primer Name	Primer Sequence (5'-3')	Comment
1.	**C4H2F (C4H2)**	TTTGGAGAAAACCCTTTTGGGTCTC	Primers used for semiquantitative PCR
2.	**C4H2R (C4H2)**	GGTACCTTCGGTTGCCGATGCAGGA	
3.	**C4H1F (C4H1)**	GTTGGAACTACAAAGAAAT	
4.	**C4H1R (C4H1)**	TTGTCCTGGTGGTGGCAAAAGC	
5.	**ActinF**	GAATGGTTAAGGCTGGATTTGCA	
6.	**ActinR**	CGGTAATTTCCTTGCTCATCCTATC	
7.	**C4HF**	GGGCCAATTCCCGTACCTAT	Primers used for Real Time PCR
8.	**C4HR**	CGAGATCAGTTAAGT TACGATGGTTT	
9.	**ActinF**	TGCACCAAGGGCTGTGTTC	
10.	**ActinR**	GCATCTTTTTGTCCCATTCCA	
11.	**GSP_Left Boarder**	CGCGCCAAAGTTCCGTCACAGGATGATCT	Gene specific primers from left and right border to analyze the site of integration.
12.	**GSP_Right Boarder**	GCCGCTGAGGAAGCTGAGTGGCGCTATTT	
13.	**GFP-Fusion -Forward**	CTCTAGAATGGATCTTCTCCTTTTGGAG	Primers used for GFP fusion
14.	**GFP-Fusion -Reverse**	CGGATCCAAAGGATCTTGGTTTAGCTACAATG	
15.	**RNAi-Sense-Forward**	CCTCGAGATGGATCTTCTCCTTTTGGAG	Primer for RNAi sense fragment
16.	**RNAi-Sense-Reverse**	CGAATTCTGCTTCCCATCCGAATCTGTA	
17.	**RNAi-Antisense-Forward**	CGGATCCATGGATCTTCTCCTTTTGGAG	Primer for RNAi antisense fragment
18.	**RNAi-Antisense-Reverse**	CAAGCTTTGCTTCCCATCCGAATCTGTA	

Table S2. Sequences of Right and left border flanking regions of the RNAi cassette integrated in the genome of RNAi plant.

Right border amplified fragment

GAATTCGATTGCCGCTGAGGAAGCTGAGTGGCGCTATTTGAAAAATCTTGAATGAATCTA
TGATAGAATCCGGCATGACCAATGAAACTCCTGACACCTTTGACAGTGGTGGTGATGAC
GTGGGATTGCGACCACGCAACAGGTTTTACCTCTCAAGGGATCTCAAGAGGCTTACAACC
CCACTAGGCAGTGTACCTGACCGATGTAGTATAGTGAAGTGGTAAGTACGGGGGATTGA
TCGTTCAACTCAAAGAATAGTGTTCGTTAGGATTGAAATTTATCTACTAGGTCTAGAGAT
GAGGGATGATTCTTTTTGTATTTTTTATGTTTTTTTGGAAAGCAAGTAAAATCTCGGGTAT
TGGAAGAGTGGGCGGAATTAGTTGCTAGTCAACTAACCCAAGAGCTACTAATTAAACTA
ATGTGCAAGAAAAGTAAATTAAGGTTCTACAAGAAAAGTGAAGCATGTCTACCTATGAT
GTATTACTCATTGCTAGCTATCCAATTCTACCTATTGACTGTAAACCTTGTTTTAATTGAC
GGGTGTATTCCTTTACCAGCCCAGGCCGTCGACCACGCGTGCCCTATAGTAATCACTAGT
GAATTC

Right border blast result

Sl No.	Discription	E-value	Query cover	Total score
**A. NCBI Blast N**				
**1**	Artemisia monosperma SRAP marker sequence	1e-42	39%	185
**2**	Mus musculus peroxisomal assembly protein PEX3P (Pex3) gene, promoter and exon 1	2e-12	8%	84.2
**3**	Solanum lycopersicum chromosome ch10, complete genome	5e-08	13%	239
**B. NCBI Blast X**				
**1**	hypothetical protein VIGAN_04092900 [Vigna angularis var. angularis]	3e-04	12%	47.4
**2**	PREDICTED: LOW QUALITY PROTEIN: uncharacterized protein LOC103326911 [Prunus mume]	6e-04	19%	50.1
**3**	PREDICTED: uncharacterized protein LOC106399145 [Brassica napus]	6e-04	12%	49.7
**C. TrichOME Database**				
**1**	EY047636 Putative non-LTR retroelement reverse transcriptase	6e-51		198

Underlined sequences are flanking genomic regions. Red sequences are GSP sequences.

Left border amplified fragment

GCCTCATATAGGGCGATTGGGCCCGACGTCGCATGCTCCCGGCCGCCATGGCGGCCGCG
GGAATTCGATTCGCGCCAAAGTTCCGTCACAGGATGATCTTCCAAATTGAGACTAACATT
CACATTACCCATTAAGACCCATGCTTCATCATTGACAATTTGTTTGAACAGGCCAAGATC
TTTCCATAATTCCCTTCTATCTCTCCCATGGTTGGCAGCATAAATGAAAGTGCAGAAGAA
CTTTGTGTTGGAGTTGAGAATTTCAATAAAATAGAACATTGCTTGCCTTGTACTATGAAT
GAGAGAGCATCTTGTCACATTGGTATTCCAACCCACAATTATTCTGCATCCCTTTACCAG
CCCGGGCCGTCGACCACGCGTGCCCTATAGTGAGTCGTATCGACCACGCGTGCCCTATAG
TGAGTCGTATTACACCACGCGTGCCCTATAGTGAGTCGTATTACACCACGCGTGCCCTAT
AGTAATCACTAGTGAATTC

Left border blast result

**A. NCBI Blast N**				
**Sl No.**	**Discription**	**E-value**	**Query cover**	**Total score**
**1**	Venerupis (Ruditapes) philippinarum DNA, microsatellite marker Asari44	1e-13	28%	210
**2**	Schistosoma mansoni SmDia gene for formin-homology protein SmDia	5e-13	27%	204
**3**	Antirrhinum majus transposon Idle-61e09b, cultivar 165E, BAC clone 165EH3_61e09	5e-13	28%	212
**B. NCBI Blast X**				
**1**	PREDICTED: uncharacterized protein LOC104899546 [Beta vulgaris subsp. vulgaris]	7e-11	62%	69.3
**2**	PREDICTED: uncharacterized protein LOC104884190 [Beta vulgaris subsp. vulgaris]	3e-09	62%	64.7
**3**	PREDICTED: uncharacterized protein LOC104898409 [Beta vulgaris subsp. vulgaris]	2e-08	61%	61.6
**C. TrichOME Database**				
**1**	No hits found			

Underlined sequences are flanking genomic regions. Red sequences are GSP sequences.

Figure S1: AaCPR expression and activity

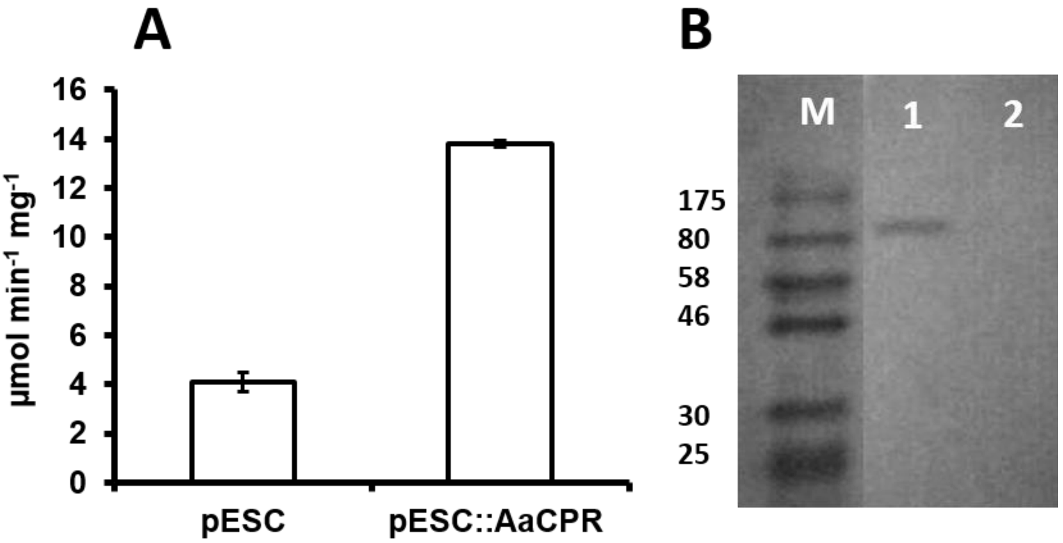

A. Reductase activity in the microsome fraction of pESC-URA vector and pESC-URA::*AaCPR* transformed *Saccharomyces cerevisiae*. Y-axis represents specific activity in µmol min-1 mg-1 protein. Specific activities were mean ± SD (n = 3 independent assays).
B. Western blot showing CPR expression in yeast microsome transformed with pESC-URA::*AaCPR* (Lane 2), pESC-URA vector (Lane 3). Protein size marker (M) is in Lane 1.

Figure S2: HPLC chromatogram showing substrate conversion by AaC4H2 (CA: *trans*-cinnamic acid; CO: *p*-coumaric acid).

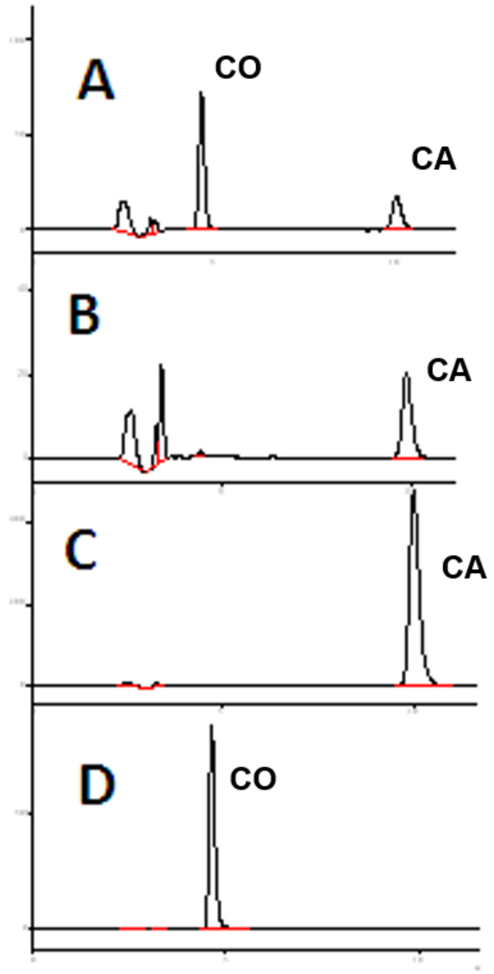

A. Conversion of *trans*-cinnamic to *p*-coumaric acid detected in the reaction product of enzyme assay using the microsome fraction of *Saccharomyces cerevisiae* transformed with pESC-URA::*AaCPR-AaC4H2*.
B. *p*-Coumaric acid was not detected in the reaction product of enzyme assay using the microsome fraction of *Saccharomyces cerevisiae* transformed with pESC-URA:: *AaCPR*.
C. *trans*-cinnamic acid standard.
D. *p*-coumaric acid standard.
